# Substitution mapping and characterization of brown planthopper resistance genes from traditional rice cultivar ‘Rathu Heenati’ (*Oryza sativa* L.)

**DOI:** 10.1270/jsbbs.23066

**Published:** 2024-07-02

**Authors:** Saw Bo Day Shar, Cuong Dinh Nguyen, Sachiyo Sanada-Morimura, Shao-Hui Zheng, Daisuke Fujita

**Affiliations:** 1 The United Graduate School of Agricultural Sciences, Kagoshima University, 1-21-24 Korimoto, Kagoshima 890-0065, Japan; 2 Loikaw Research Center, Department of Agricultural Research, Loikaw 09011, Kayah State, Myanmar; 3 Biotechnology Department, College of Food Industry, 101B Le Huu Trac Street, Son Tra District, Da Nang City 550000, Vietnam; 4 Koshi Campus, Division of Core Technology for Pest Control Research, Institute for Plant Protection, NARO, 2421 Suya, Koshi, Kumamoto 861-1192, Japan; 5 Faculty of Agriculture, Saga University, 1 Honjo-machi, Saga 840-8502, Japan

**Keywords:** rice, BPH resistance, gene mapping, pyramiding

## Abstract

The brown planthopper (BPH; *Nilaparvata lugens* Stål) is a devastating pest that causes severe rice yield losses in Asia. Introducing multiple BPH resistance genes into rice cultivars is an effective and sustainable way to mitigate yield losses. A traditional rice cultivar, ‘Rathu Heenati’, has durable BPH resistance due to multiple resistance genes (including *BPH3* and *BPH17*) and quantitative trait loci (QTLs). However, these genes have not been used in Japanese rice breeding owing to limited genetic information. To identify markers tightly linked to *BPH3* and *BPH17* introgressed into the ‘Sagabiyori’ (susceptible) genetic background, we performed substitution mapping. *BPH3* was delimited between RM3132 and RM589 on chromosome 6, and *BPH17* between RM16493 and RM16531 on chromosome 4. We also performed QTL analysis to identify additional BPH resistance genes from ‘Rathu Heenati’ and detected a QTL, denoted as *qBPH3.1*, on chromosome 3. The effect of pyramiding *BPH3* and *BPH17* was significantly greater against virulent BPH populations than that of either gene alone. The combination of *BPH3*, *BPH17* and *qBPH3.1* from ‘Rathu Heenati’ might be facilitated to improve commercial Japanese cultivars with more robust BPH resistance.

## Introduction

Rice (*Oryza sativa* L.) is one of the most important crops in the Asia–Pacific region and is host to a wide range of insect pests. The brown planthopper (BPH), *Nilaparvata lugens* Stål, is a destructive insect pest of rice and causes severe damage by feeding on phloem sap and transmitting serious viral diseases such as *Rice grassy stunt virus* (RGSV) and *Rice ragged stunt virus* (RRSV) (Fujita *et al.* 2013, Wei *et al.* 2018). Extremely high BPH population levels in rice fields cause “hopper burn” and, consequently, yield losses of nearly $300 million annually in Asia (Min *et al.* 2014). In China, more than 25 million hectares were affected by BPH from 2005 to 2008, resulting in the loss of about 3 million tons of rice yield (Hu *et al.* 2016, Qiu *et al.* 2012) and approximately 0.57 million hectares of rice in Vietnam was devastated in 2007 (Catindig *et al.* 2009). BPH damaged over 3 million hectares of rice fields in Thailand between 2009 and 2011, and as much as 0.2 million hectares of rice in Indonesia in 2011 (Horgan *et al.* 2015). Furthermore, BPH outbreaks in 2013 and 2019 in western and southwestern Japan led to substantial economic losses of 10.5 × 10^9^ JPY in rice production in 2013 and comparable losses in 2019 (Sanada-Morimura 2020).

For several decades, the improvement of host plant resistance in rice has been an effective and environmentally friendly approach to reducing BPH damage (Khush 2001). To date, at least 45 loci for BPH resistance (designated as *BPH1* to *BPH45*) have been identified and mapped across all 12 chromosomes of rice (Du *et al.* 2020, Fujita *et al.* 2013, Wang *et al.* 2022, Yang *et al.* 2019). Groups of three or more loci that are closely positioned (e.g., within a 5- to 10-Mbp region) or overlap within the same chromosome region are known as clusters. In the case of BPH resistance, five clusters have been designated: cluster A (chromosome 12), cluster B (chromosome 4S), cluster C (chromosome 6S), cluster D (chromosome 4L) and cluster E (chromosome 3L) (Fujita *et al.* 2013, Hu *et al.* 2016). Although the known BPH resistance genes remain effective against BPH populations with lower virulence, many genes have been overcome by specific BPH populations. Moreover, the virulence of BPH populations against resistant cultivars progressively increases over time (Fujii *et al.* 2021, Myint *et al.* 2009a). However, resistance genes with less effectiveness against highly virulent BPH can still be useful for combining with other BPH resistance genes to enhance resistance levels (Myint *et al.* 2012). Therefore, it remains crucial to understand the genetic basis and resistance mechanism of resistance genes, even if they confer relatively low resistance against highly virulent BPH populations.

‘Rathu Heenati’, a traditional cultivar from Sri Lanka, carries multiple resistance genes, including *BPH3*, *BPH17*, *BPH14*, *Qbph3* and *Qbph10* (Jairin *et al.* 2007b, Pannak *et al.* 2023, Sun *et al.* 2005), and has been used as a BPH-resistant control for decades (Fujii *et al.* 2021, Horgan *et al.* 2015). Two of them, *BPH3* and *BPH17*, were detected on chromosomes 6 and 4, respectively (Jairin *et al.* 2007a, Sun *et al.* 2005). *BPH3* from ‘Rathu Heenati’ was mapped between RM19291 and RM8072 on the short arm of chromosome 6 (Jairin *et al.* 2007a, 2007b). The position of *BPH3* overlapped with that of *BPH32* on chromosome 6 (Jairin *et al.* 2007c, Ren *et al.* 2016). Sun *et al.* (2005) identified *BPH17* on chromosome 4, which is the same as *BPH3* reported by Liu *et al.* (2015). As *BPH3* (“*BPH17*” was used based on Sun *et al.* (2005) in this study) encodes plasma membrane–localized lectin receptor kinases OsLecRK1–3, this suggests that the three genes in a cluster (*OsLecRK1–3*) contribute to durable BPH resistance (Liu *et al.* 2015). There are SSR and insertion-deletion (InDel) markers linked to *BPH3* (“*BPH17*” in this study) (Liu *et al.* 2015) that have been reported and utilized for both *indica* and *japonica* backgrounds. However, *BPH17* exhibits high allele variation (347 variations), including rare alleles, and limited information is available for SNPs related to BPH resistance. Therefore, He *et al.* (2020) developed eight InDel markers (located at 6.9–7.0 Mbp around *BPH17*) linked to BPH resistance. The verification of co-segregation between DNA markers and BPH resistance facilitates the effective utilization of co-segregated markers in transferring BPH resistance genes into the genetic background of elite cultivars, mitigating the risk of introducing linkage drag (Jena and Mackill 2008).

Previous studies have revealed that rice cultivars carrying multiple resistance genes had stronger resistance than those carrying a single resistance gene (Fujii *et al.* 2021, Myint *et al.* 2009b). Pyramided lines (PYLs) containing *BPH3* and *BPH17* (*BPH3+17*) from ‘Rathu Heenati’ in the genetic backgrounds of susceptible *japonica* cultivar ‘Taichung 65’ (T65) and resistant *indica* cultivar ‘IR64’ have been developed and characterized for BPH resistance (Kamal *et al.* 2023, Nguyen *et al.* 2019). However, the resistance levels and effectiveness of gene pyramiding on current commercial Japanese cultivars susceptible to BPH have remained unclear. Furthermore, the resistance level of T65-*BPH3+17* was higher than that of either T65-*BPH3* or T65-*BPH17* but significantly lower than that of ‘Rathu Heenati’, suggesting that ‘Rathu Heenati’ might carry other unknown genetic factors related to BPH resistance.

Identification and mapping of BPH resistance genes have contributed not only to elucidating their resistance mechanisms but also to the introduction of resistance genes through the use of MAS. The commercial *japonica* rice cultivar ‘Sagabiyori’ possesses excellent heat tolerance and high grain quality along with a significant amount of soluble starch in its stem. Phloem-sucking by BPH, which takes soluble starch from the leaf sheath and disrupts its translocation, may lead to hopper burn (Watanabe and Kitagawa 2000). Owing to the susceptibility of ‘Sagabiyori’ to BPH damage, there is an urgent requirement to increase its BPH resistance. To do so, near-isogenic lines (NILs) for *BPH3* (Saga-*BPH3*) and *BPH17* (Saga-*BPH17*) have been developed (Shar *et al.* 2023). However, the intervals of flanking markers for *BPH3* and *BPH17* were large and we have limited information on markers tightly linked to *BPH3* and *BPH17* within the ‘Sagabiyori’ genetic background. Therefore, to identify markers closely linked to *BPH3* and *BPH17* within the ‘Sagabiyori’ genetic background, we performed substitution mapping using homozygous recombinant lines derived from the corresponding NILs. Additionally, the resistance levels of Saga-*BPH3* and Saga-*BPH17* weren’t high against the BPH population that has currently migrated into Japan. To clarify the effect of pyramiding *BPH3* and *BPH17* within the ‘Sagabiyori’ background, we characterized the effectiveness of Saga-*BPH3+17* against current virulent BPH populations. Furthermore, to identify BPH resistance genes other than *BPH3* and *BPH17*, we developed a backcross population derived from T65 × ‘Rathu Heenati’ and conducted QTL analysis.

## Materials and Methods

### Plant materials for substitution mapping, pyramided line and QTL analysis

NILs for *BPH3* and *BPH17* from ‘Rathu Heenati’ (accession number IRGC 11730) in the ‘Sagabiyori’ genetic background were developed from BC_3_ progeny by Shar *et al.* (2023). To conduct substitution mapping using BC_3_ progeny, we screened recombinants around *BPH3* and *BPH17* using 384 plants from each BC_3_F_2_ population segregating for resistance at *BPH3* or *BPH17*. From the BC_3_F_3_ lines, we selected homozygous recombinant lines by using SSR markers flanking *BPH3* or *BPH17*. In BC_3_F_4_ lines, plants homozygous for recombination events around *BPH3* or *BPH17* were used for evaluation of BPH resistance.

To characterize the effect of pyramiding *BPH3* and *BPH17*, we developed a PYL containing both resistance alleles by crossing BC_3_F_2_ plants with *BPH3* (Saga-*BPH3*) and BC_3_F_2_ plants with *BPH17* (Saga-*BPH17*) (Supplemental Fig. 1). The F_1_ was self-pollinated to produce an F_2_ population, and plants homozygous for resistance alleles of both *BPH3* and *BPH17* were selected from the F_2_ population by MAS. Finally, the selected F_2_ plants were self-pollinated to produce F_3_ seed, and F_3_ plants were used for evaluation of the resistance.

To identify loci for BPH resistance other than *BPH3* and *BPH17*, we crossed T65 (as a susceptible parent) with ‘Rathu Heenati’ to develop F_1_ plants. The F_1_ plants were backcrossed with T65, and BC_1_F_1_ plants without *BPH3* and *BPH17* alleles from ‘Rathu Heenati’ were selected by MAS. The selected BC_1_F_1_ plants were self-pollinated to develop BC_1_F_2_ lines. Among the BC_1_F_2_ lines, those with BPH resistance were selected, and 82 BC_1_F_2:3_ lines (RHH1–RHH82) were used to conduct QTL analysis (Supplemental Fig. 2). NILs and a PYL carrying BPH resistance genes in the T65 genetic background (T65-*BPH3*, T65-*BPH17* and T65-*BPH3+17*) were developed by Nguyen *et al.* (2019) and used here for comparison to study the mechanisms of resistance conferred by the identified QTL (see Results).

### DNA extraction and genotyping

Total DNA from backcross lines was extracted by the potassium acetate method (Dellaporta *et al.* 1983). The genotypes of SSR markers in plants in each generation were determined by polymerase chain reaction (PCR) and agarose gel electrophoresis as described by Shar *et al.* (2023). In the BC_3_F_2_ population segregating at *BPH3*, plants with recombination events around *BPH3* were selected by using four SSR markers on chromosome 6S: RM6775, RM508, RM588 and RM19341. In the BC_3_F_2_ population segregating at *BPH17*, recombinants were selected by using four DNA markers on chromosome 4S (RM8213, RM1305, RM16531 and B40). The BC_3_F_4_ homozygous recombinant lines for *BPH3* were genotyped with 17 additional SSR markers between RM6775 and RM19341 (Supplemental Table 1). Similarly, the BC_3_F_4_ homozygous recombinant lines for *BPH17* were genotyped with 8 additional DNA markers between RM8213 and B40. Additionally, InDel marker, BPH32 dete 1, was developed on the exon of *BPH32* based on 9-bp nucleotide sequence difference between ‘Sagabiyori’ and Saga-*BPH32*. Moreover, two InDel markers I531 and I729 (He *et al.* 2020), those are tightly linked to *BPH17* region, were used to confirm co-segregation with *BPH17* on the BC_3_F_4_ recombinant lines (Supplemental Table 1).

### BPH population used for evaluation of plant resistance

The BPH population collected in Hadano city, Kanagawa, Japan, in 1966 (Hadano-1966), showed no virulence to any BPH resistant genes because it was collected before resistant varieties were widely distributed (Myint *et al.* 2009b). Hadano-1966 (biotype1) was used for evaluating resistance in substitution mapping and characterization of resistance mechanisms for *qBPH3.1*. For the evaluation of PYLs and corresponding NILs, two BPH populations with strong virulence were used. Koshi-2013 was collected in Koshi city, Kumamoto, Japan, in 2013 (Fujii *et al.* 2021), and Koshi-2020 was collected in 2020. In previous long-term virulence monitoring of BPH on differential resistant varieties, Koshi-2013 exhibited virulence to several resistant varieties: ‘Mudgo’ (*BPH1*), ‘ASD7’ (*BPH2*) and ‘Babawee’ (*BPH4*) (Fujii *et al.* 2021) and it was not possible to classify specific biotype based on existing definitions. These populations were maintained on the susceptible *japonica* cultivar ‘Reiho’ at 25°C under 16 h light/8 h dark at NARO, Kumamoto, Japan, and were provided to Saga University. At Saga University, all strains were maintained separately on T65 under the above conditions.

### Modified seedbox screening test and modified mass tiller screening

A modified seedbox screening test (MSST) was conducted to evaluate the resistance levels of BC_3_F_4_ homozygous recombinant lines for *BPH3* and *BPH17*, Saga-*BPH3* and Saga-*BPH17*, Saga-*BPH3+17* and a population for QTL analysis. The MSST was performed as described by Shar *et al.* (2023), with minor modifications. Here, we infested seedlings with BPH nymphs at 7 days after sowing (DAS). When the susceptible cultivar was dead, we scored the materials on a scale of 0 (no damage) to 9 (dead) following the standard damage score (DS) evaluation for rice (IRRI 2014).

Modified mass tiller screening (MMTS), a method described by Jairin *et al.* (2007b), was used for the evaluation of homozygous recombinant lines for *BPH3*. Seeds of each line and of ‘Rathu Heenati’ and ‘Sagabiyori’ were separately sown in 1-L pots. At 60 DAS, tillers with similar growth stage were separated and transplanted into a plastic box (70.0 cm × 45.0 cm × 60.0 cm). At ten days after transplanting, the plants were infested with second- and third-instar BPH nymphs at a density of approximately 40 nymphs per tiller. At 7 days after infestation (DAI), the DS values of the homozygous recombinant lines and parents were evaluated.

### Antibiosis test

Antibiosis tests were conducted at 25°C as described by Myint *et al.* (2009b), with minor modifications. Seeds of Saga-*BPH3*, Saga-*BPH17* and Saga-*BPH3+17* were individually sown in 215-mL plastic cups. Seven- and thirty-day-old seedlings were infested, and the adult mortality (ADM) and swollen-abdomen percentage (SA) of infested female BPH adults were observed at 5 DAI. The abdomen size of female BPHs was classified as small, medium and large according to nutrient intake, and the SA was calculated as the ratio of medium and large. We also conducted resistance mechanism tests at the BC_1_F_3_ generation as described above.

To study the effects of the identified QTL (*qBPH3.1+*), the rates of BPH feeding on the BC_1_F_3_ lines were determined as described by Heinrichs *et al.* (1985) with minor modifications. Each BC_1_F_3_ lines (*qBPH3.1+*), T65-*BPH3*, T65-*BPH17* and T65-*BPH3+17* were individually sown in 215-mL plastic cups. In this test, we infested 7- and 30-day-old seedlings and measured the area of honeydew excreted from female BPH adults after feeding on plants for 24 h.

### Antixenosis test

One plant from each line carrying *qBPH3.1* (*qBPH3.1+*) or without *qBPH3.1* (*qBPH3.1–*) was sown together with T65 in a 215-mL plastic cup, with five replicates. At 30 DAS, the plants in each cup were covered with plastic tubes with ventilators. Into each tube, we placed 20 second-instar BPH nymphs. The number of insects that settled on each plant was recorded every day until 5 DAI. The antixenosis level was calculated as the percentage of insects settled on each plant out of the 20 total placed into each tube.

### QTL analysis

The initial population selection was conducted on 36 BC_1_F_2_ lines derived from a cross between T65 and ‘Rathu Heenati’. This selection was designed to identify resistant BC_1_F_2_ lines lacking *BPH3* and *BPH17* through MSST and MAS (Supplemental Fig. 3). A BC_1_F_2:3_ population with BPH resistance (N = 82), that has homozygous for the T65 alleles of *BPH3* and *BPH17*, was chosen for QTL analysis. For genotyping, among 384 SSR markers evenly distributed across the 12 rice chromosomes, we used 30 SSR markers that exhibited polymorphic between T65 and the resistance BC_1_F_2_ (bulked). Regarding phenotyping, we assessed the BC_1_F_3_ lines (82 lines) by MSST. To detect QTLs for BPH resistance in the BC_1_F_2:3_ population, we performed composite interval mapping using Windows QTLs Cartographer v. 2.5 software. The threshold value for the logarithm of odds (LOD) score was 2.0 based on a 1000-permutation test at *P* < 0.05.

### Statistical analysis

Mean values of DS for the substitution mapping and QTL-carrying BC_1_F_3_ lines, ADM, SA and honeydew area of NILs, PYLs, BC_1_F_3_ lines and parental lines were compared by one-way ANOVA. The Tukey–Kramer test was used for multiple comparisons of resistance level by MSST, antibiosis tests and honeydew area of NILs, PYLs, BC_1_F_3_ lines and parental lines. Student (*t*-test) was applied to compare the antixenosis level between lines and T65. Dunnett’s test was used for the multiple comparisons of *BPH3* and *BPH17* homozygous recombinant lines against ‘Sagabiyori’ in R v. 4.1.2 software.

## Results

### Substitution mapping of *BPH3* and *BPH17*

**Fig. 1.**
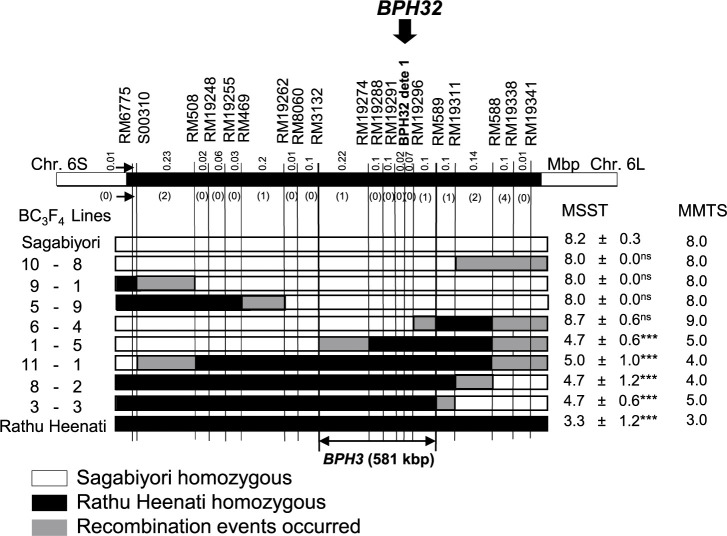
Substitution map of *BPH3* on chromosome 6. The labels at the top indicate the physical positions of DNA markers around the location of *BPH3* on chromosome 6. *BPH32* with arrow head indicate the location of *BPH32* from ‘Ptb33’. Vertical lines indicate the positions of these DNA markers in the tested parental and BC_3_F_4_ lines. The numbers above the top bar indicate the physical distances between the markers, and the numbers in parentheses below the bar indicate the number of recombinants within each interval. ***Significant difference from damage score of ‘Sagabiyori’ (*P* < 0.001, Dunnett’s multiple comparison test against ‘Sagabiyori’); ns, no significant difference. MSST, modified seedbox screening test (values are mean ± standard deviation); MMTS, modified mass tiller screening.

To delimit the locations of BPH resistance genes *BPH3* and *BPH17* from ‘Rathu Heenati’, we used BC_3_F_4_ lines for substitution mapping. Among 384 BC_3_F_2_ plants segregating at *BPH3*, we identified 22 plants carrying recombination events that occurred between two flanking markers, RM6775 and RM19341, on chromosome 6. Using additional markers in this region, we selected eight lines with different homozygous recombinant segments from ‘Rathu Heenati’ (Fig. 1) and evaluated their BPH resistance against Hadano-1966 using MSST and MMTS. By MSST, DS of ‘Sagabiyori’ was 8.2 and that of ‘Rathu Heenati’ was 3.3 (Fig. 1). Among the eight selected BC_3_F_4_ lines, three (5-9, 9-1 and 10-8) that were homozygous for ‘Sagabiyori’ alleles at all marker loci between RM19262 and RM19311 were susceptible to BPH by both MSST and MMTS. In contrast, three lines (3-3, 8-2 and 11-1) homozygous for ‘Rathu Heenati’ alleles at all marker loci between RM508 and RM589 were resistant to BPH by both MSST and MMTS. Line 6-4 (DS = 8.7 [MSST] and 9.0 [MMTS]), which was homozygous for ‘Sagabiyori’ alleles between RM6775 and RM19296, was susceptible. Line 1-5, which was homozygous for ‘Rathu Heenati’ alleles between RM19274 and RM588, was resistant. Therefore, *BPH3* was delimited between RM3132 and RM589 on chromosome 6, an interval of ~581 kbp on the ‘Nipponbare’ genome sequence (Fig. 1). The InDel marker, BPH32 dete 1, co-segregated with *BPH3* in the BC_3_F_4_ recombinant lines (Fig. 1).

**Fig. 2.**
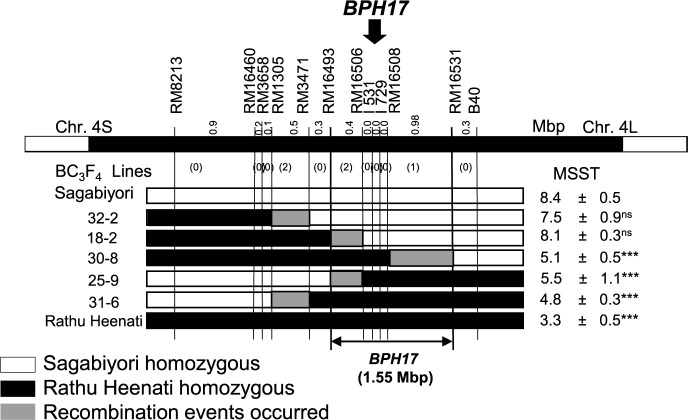
Substitution map of *BPH17* on chromosome 4. The labels at the top indicate the physical positions of DNA markers around the location of *BPH17* on chromosome 4. *BPH17* with arrow head indicate the location of *BPH17*. Vertical lines indicate the positions of these DNA markers in the tested parental and BC_3_F_4_ lines. The numbers above the top bar indicate the physical distances between markers and the numbers in parentheses indicate the number of recombinants within each interval. ***Significant difference from damage score of ‘Sagabiyori’ (*P* < 0.001, Dunnett’s multiple comparison test against ‘Sagabiyori’); ns, no significant difference. MSST, modified seedbox screening test (values are mean ± standard deviation).

In the population segregating at *BPH17*, 23 of 384 BC_3_F_2_ plants carried recombination events that occurred between RM8213 and B40 on chromosome 4. From these, we selected five lines with homozygous ‘Rathu Heenati’ overlapping *BPH17* regions (Fig. 2) and evaluated them for BPH resistance against Hadano-66 by MSST. Lines 18-2 and 32-2, both of which were homozygous for a ‘Sagabiyori’ segment between RM16506 and B40, were susceptible to BPH. The DS values of lines 18-2 (8.1) and 32-2 (7.5) were not significantly different from that of ‘Sagabiyori’ (8.4) (Fig. 2). Lines 25-9 and 31-6, homozygous for ‘Rathu Heenati’ segments between RM16506 and B40, and line 30-8, homozygous for a ‘Rathu Heenati’ segment between RM8213 and RM16508, showed resistance to BPH. The DS values of the three lines (all ≤5.5) were significantly lower than that of ‘Sagabiyori’. These results place *BPH17* between RM16493 and RM16531 on chromosome 4, an interval of ~1.55 Mbp on the ‘Nipponbare’ genome sequence. Two InDel markers, I531 and I729, co-segregated with *BPH17* in the BC_3_F_4_ recombinant lines (Fig. 2).

### Characterization of resistance levels of pyramided line

**Fig. 3.**
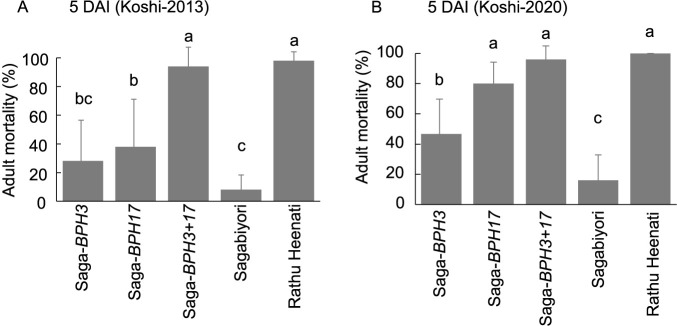
Adult mortality of BPH on *BPH3* and *BPH17* NILs and on the *BPH3+BPH17* PYL at 5 days after infestation with (A) Koshi-2013 and (B) Koshi-2020 BPH populations in an antibiosis test. Means labeled with the same letter do not differ significantly at *P* < 0.01 by the Tukey–Kramer test.

In antibiosis, ADM on PYL carrying *BPH3* and *BPH17* resistance alleles in the ‘Sagabiyori’ genetic background (Saga-*BPH3+17*) was evaluated to compare with that on Saga-*BPH3* and Saga-*BPH17* infested with Koshi-2013 and Koshi-2020 (Fig. 3). When infested with Koshi-2013, ADM was significantly higher on Saga-*BPH3+17* (94%) than on Saga-*BPH3* (28%), Saga-*BPH17* (38%) and ‘Sagabiyori’ (8%). When infested with Koshi-2020, ADM was significantly higher on Saga-*BPH3+17* (96%) than on Saga-*BPH3* (47%) and ‘Sagabiyori’. There was no significant difference in ADM of Koshi-2020 between Saga-*BPH17* (80%) and Saga-*BPH3+17* (96%). Thus, it can be assumed that the high resistance level of Saga-*BPH3+17* was mostly due to the effect of *BPH17*.

**Fig. 4.**
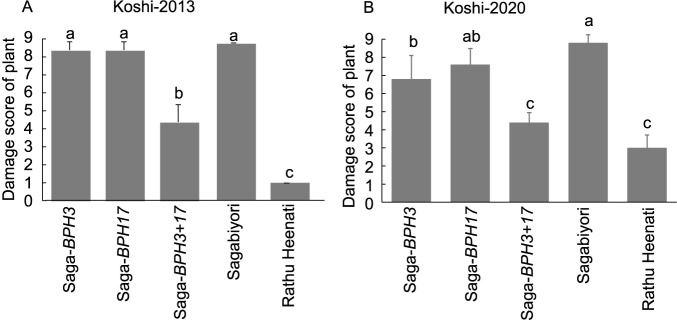
Damage scores of NILs and PYL for *BPH3* and *BPH17* infested with Koshi-2013 (A) and Koshi-2020 (B) in a modified seedbox screening test (MSST). Values marked with the same letter do not differ significantly at *P* < 0.05 by the Tukey–Kramer test.

In the MSST, when infested with Koshi-2013, DS values of Saga-*BPH3+17* (4.5) and ‘Rathu Heenati’ (1.0) were significantly lower than those of Saga-*BPH3* (8.6), Saga-*BPH17* (8.6) and ‘Sagabiyori’ (9.0) (Fig. 4A). When infested with Koshi-2020, DS values of Saga-*BPH3+17* (4.4) and ‘Rathu Heenati’ (3) were significantly lower than those of Saga-*BPH3* (6.8), Saga-*BPH17* (7.6) and ‘Sagabiyori’ (8.8) (Fig. 4B). Therefore, pyramiding of *BPH3* and *BPH17* enhanced the resistance level of ‘Sagabiyori’ against the Koshi-2013 and Koshi-2020 BPH strains. However, against Koshi-2013, DS of *BPH3+17* was significantly higher than that of ‘Rathu Heenati’, suggesting that ‘Rathu Heenati’ might carry other genetic factors for BPH resistance.

### Detection of QTL for BPH resistance from ‘Rathu Heenati’

To identify loci for BPH resistance from ‘Rathu Heenati’ other than *BPH3* and *BPH17*, we conducted QTL analysis using BC_1_F_2:3_ population that did not carry *BPH3* or *BPH17* from ‘Rathu Heenati’. Firstly, DS values of 36 BC_1_F_2_ lines against Hadano-1966 by MSST were evaluated and selected BPH resistant lines. Through MAS, we selected a BPH-resistant line that did not carry *BPH3* and *BPH17* from ‘Rathu Heenati’ to develop a BC_1_F_2_ population for QTL analysis (Supplemental Fig. 3). In the BC_1_F_2:3_ population (N = 82), the frequency distribution of DS was continuous (Supplemental Fig. 4). The graphical genotype of the BC_1_F_1_ plant was estimated by bulked BC_1_F_2_ plants and polymorphic markers on heterozygous regions were used for genotyping of BC_1_F_2_ population (Supplemental Fig. 5). By QTL analysis, a single QTL for BPH resistance was detected between RM16209 and RM16231 on the long arm of chromosome 3 with a proportion of phenotypic variance of 26.2%, and the detected QTL was denoted as *qBPH3.1* (Table 1, Supplemental Fig. 6). The additive effect of ‘Rathu Heenati’ allele of *qBPH3.1* reduced DS and increased BPH resistance. Most plants with ‘Rathu Heenati’ homozygous for RM16209 near *qBPH3.1* was low DS, while the plants with T65 homozygous was high DS (more than 6) (Supplemental Figs. 4, 7).

### Characterization of resistance mechanisms of *qBPH3.1*

**Fig. 5.**
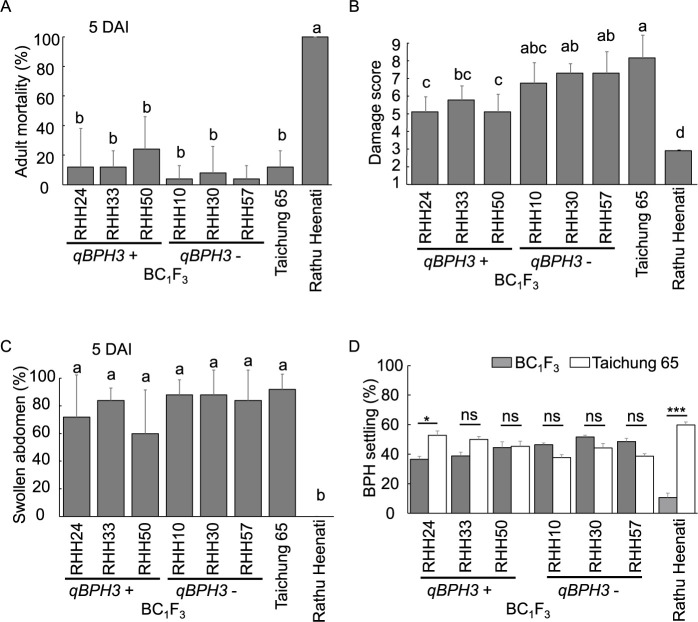
Evaluation of resistance mechanisms on BC_1_F_3_ lines carrying *qBPH3.1* (*qBPH3.1+*) and without *qBPH3.1* (*qBPH3.1*–). (A) Adult mortality (%); (B) damage score (assessed by MSST); (C) swollen abdomen (%); and (D) BPH settling (%). Values in A–C marked with the same letter are not significantly different according to the Tukey–Kramer test at *P* < 0.05. Asterisks in (D) indicate significant difference between the indicated line and ‘Taichung 65’: * *P* < 0.05, *** *P* < 0.001 by *t*-test. DAI = days after infestation.

**Fig. 6.**
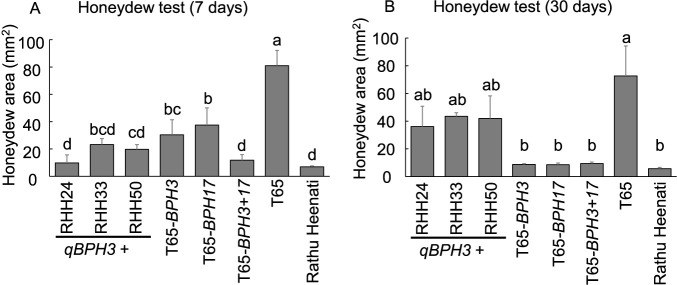
Feeding activity evaluation of *qBPH3.1*-carrying BC_1_F_2:3_ lines (RHH24, RHH33 and RHH50), T65-*BPH3*, T65-*BPH17* and T65-*BPH3+17*, with ‘Taichung 65’ genetic background. Honeydew area on seedlings inoculated at 7 days old (A) and 30 days old (B), determined 24 h after infestation. Values marked with the same letter are not significantly different according to the Tukey–Kramer test at *P* < 0.05.

To characterize the resistance mechanisms of *qBPH3.1*, we evaluated BC_1_F_3_ lines against Hadano-1966 by MSST, honeydew tests, antibiosis tests and antixenosis tests (Figs. 5, 6). The DS values of two of three lines homozygous for the ‘Rathu Heenati’ allele of *qBPH3.1* (*qBPH3.1+*: RHH24 and RHH50) were significantly lower than those of two of three lines homozygous for the T65 allele (*qBPH3.1–*: RHH30 and RHH57) and T65 (Fig. 5B). However, there were no significant differences in ADM, swollen abdomen percentage or BPH settling percentage (antixenosis) between *qBPH3.1+* and *qBPH3.1–* lines (Fig. 5A, 5C). With the exception of RHH24, there were no significant differences in settling percentage between *qBPH3.1+* and *qBPH3.1–* lines and the T65 control (Fig. 5D). In addition, we compared the feeding activity of BPH at 7 and 30 DAS by using a honeydew test including three *qBPH3.1+* lines as well as T65-*BPH3*, T65-*BPH17* and T65-*BPH3+17* (Fig. 6). At the 7-day-old seedling stage, the honeydew areas of *qBPH3.1+* lines RHH24 (9.8 mm^2^), RHH33 (23.2 mm^2^) and RHH50 (19.9 mm^2^) were marginally lower than those of T65-*BPH3* (30.4 mm^2^) and T65-*BPH17* (37.5 mm^2^), with some of the differences being statistically significant (Fig. 6A). The honeydew area of each *qBPH3.1+* line was significantly lower than that of T65 (80.9 mm^2^), suggesting that BPH feeding was inhibited (Fig. 6A). In contrast, the honeydew areas of *qBPH3.1+* lines at the 30-day-old seedling stage—RHH24 (36.0 mm^2^), RHH33 (43.5 mm^2^) and RHH50 (41.9 mm^2^)—were higher (although not significantly) than those of T65-*BPH3* (8.8 mm^2^) and T65-*BPH17* (8.6 mm^2^), and lower (although not significantly) than that of T65 (Fig. 6B). Hence, the resistance level conferred by *qBPH3.1* against BPH feeding was high in 7-day-old seedlings but lower in 30-day-old seedlings.

## Discussion

In the current rice cultivation in Japan, there are issues with reduction of grain yield and quality due to high temperature exposure during ripening period of Japanese cultivars (Tanaka *et al.* 2009). Thus, heat tolerance rice varieties with high non-structural carbohydrates (NSC) content in the stem, such as ‘Sagabiyori’, have been developed to maintain rice grain quality (Tanamachi *et al.* 2016). However, ‘Sagabiyori’ with high NSC content was found to be highly susceptible to BPH. In previous study, to enhance BPH resistance in ‘Sagabiyori’, seven NILs for BPH resistance genes with the genetic background of ‘Sagabiyori’—Saga-*BPH2*, Saga-*BPH17-ptb*, Saga-*BPH32* (from ‘Ptb33’), Saga-*BPH3*, Saga-*BPH17* (from ‘Rathu Heenati’), Saga-*BPH20* and Saga-*BPH21* (from ‘IR71033-121-15’)—were developed and characterized (Shar *et al.* 2023). Among the seven BPH resistance genes, the NILs for *BPH3* or *BPH17* exhibited moderate resistance to BPH population (Koshi-2013). Therefore, our focus was on the *BPH3* and *BPH17* to identify markers that co-segregating with BPH resistance genes through substitution mapping and the co-segregating markers could facilitate MAS in rice breeding in Japan.

The region of *BPH3* on short arm of chromosome 6 was clustered with other genes: *BPH4*, *BPH25*, *BPH29* and *BPH32* (Fujita *et al.* 2013, Mishra *et al.* 2022). Shar *et al.* (2023) reported that the resistant level of Saga-*BPH3* was higher than Saga-*BPH32* in most resistant tests. It proposed the possibility that Saga-*BPH3* might be related to other genetic factors or different alleles due to the clustered region on short arm of chromosome 6. Through substitution mapping in this study, we delimited a 581 kb region for *BPH3* as a single genetic factor. This region still overlapped with the location of *BPH32* (Fig. 1). The InDel marker, BPH32 dete 1 was found to be co-segregated with *BPH3* among BC_3_F_4_ recombinant lines (Fig. 1, Supplemental Fig. 8). This InDel marker will be useful for efficiently selecting *BPH3* on chromosome 6 by MAS. Additionally, Jairin *et al.* (2007b) previously reported that *BPH3* was mapped on chromosome 6 through MMTS at 60-day-old plant stage and exhibited unstable resistance at seedling stage. In our study, by using Hadano-1966, *BPH3* showed resistance at 7-day-old plant stage through MSST and at 60-day-old plant stage through MMTS (Fig. 1). This result suggests that *BPH3* can maintain BPH resistance from the seedling to the tillering stages in the ‘Sagabiyori’ genetic background.

The amino acid sequences of *BPH3* (“*BPH17*” was used in this study) from ‘Rathu Heenati’ were found to be identical to those of *BPH17-ptb* (from ‘Ptb33’) (Liu *et al.* 2015). However, Saga-*BPH17* exhibited higher resistance level than Saga-*BPH17-ptb* in several tests (Shar *et al.* 2023). The observed difference in BPH resistance and presence of a clustered region on short arm of chromosome 4 may suggest the involvement of another genetic factor in *BPH17* region or the presence of different alleles. Through substitution mapping in this study, we delimited a 1.55 Mbp region for *BPH17* as a single genetic factor on chromosome 4 but we were unable to elucidate the reason for the differing resistance level (Fig. 2). Furthermore, He *et al.* (2020) developed eight InDel markers on *BPH17* locus to distinguish between resistant and susceptible alleles by comparing genomic sequences of 50 rice varieties carrying three *OsLecRK* genes. Out of these eight InDel markers, I531 and I729 were found to be co-segregated with *BPH17* in the segregating population of the ‘Sagabiyori’ genetic background (Fig. 2, Supplemental Fig. 8). Therefore, these two InDel markers, those co-segregated with *BPH17* in this study, will be valuable for future MAS breeding of Japanese rice cultivars.

According to Fujii *et al.* (2021), the virulence of BPH that has migrated into Japan has been strengthening year by year. Koshi-2013 is unable to survive on resistance varieties ‘Balamawee’ (*BPH27* and three QTLs) and ‘Rathu Heenati’ (*BPH3*, *BPH17*, *BPH14* and two *QTLs*), while BPH in 2019 can survive on ‘Rathu Heenati’. Therefore, different BPH populations were used in this study to confirm the effectiveness of BPH resistance genes against the latest collected BPH population. Our study revealed that Saga-*BPH3+17* exhibited a higher level of resistance against virulent populations, Koshi-2013 and Koshi-2020 (Figs. 3A, 4A, 4B). In Kamal *et al.* (2023), the PYLs for *BPH3* and *BPH17* in the ‘IR64’ genetic background (carrying *BPH1* and *BPH37*) also showed resistance against Koshi-2013 and Koshi-2020 (Yang *et al.* 2019). Although commercial Japanese cultivar ‘Sagabiyori’ is heat tolerance with high NSC content and highly susceptible to BPH, the introduction of *BPH3* and *BPH17* significantly increased the BPH resistant level through gene pyramiding. Currently developed Japanese rice varieties, such as ‘Genkitsukushi’ and ‘Nikomaru’, are also heat tolerance and have high NSC content (Tanamachi *et al.* 2016). Therefore, introducing *BPH3* and *BPH17* could enhance BPH resistance in these rice varieties with heat tolerance. The utilization of *BPH3* and *BPH17* could contribute to preventing the BPH outbreaks, such as those observed in 2013 and 2019 in Japan, and enhance BPH resistance in Japanese rice varieties with heat tolerance.

Despite the enhancement in BPH resistance by two-gene pyramiding, the resistance level of ‘Rathu Heenati’ was higher than that of Saga-*BPH3+17* (Figs. 3, 4). Consequently, we estimated that the durable BPH resistance of ‘Rathu Heenati’ involves another genetic factor beyond *BPH3* and *BPH17*. Through QTL analysis using BC_1_F_2:3_, we identified a single QTL, *qBPH3.1* (Table 1), near the *BPH14* region on chromosome 3 (Du *et al.* 2009). We evaluated the resistance mechanisms of *qBPH3.1* using various tests (Figs. 5, 6). Our results suggest that the resistance conferred by *qBPH3.1* was higher in young seedlings (Fig. 5). Additionally, the effect of sucking inhibition of *qBPH3.1* in young seedlings (7-day-old) was investigated through honeydew test (Fig. 6A, 6B). The resistance mechanism of *qBPH3.1* inferred from our results was similar to that observed in a previous study of *BPH14*-containing transgenic plants, which prevented the ingestion stylet into the phloem and reduced the honeydew area at the four-leaf stage (Du *et al.* 2009). Pannak *et al.* (2023) also investigated the BPH resistance of ‘Rathu Heenati’ at early growth stages attributed to the presence of functional *BPH14*. Our results suggest that the robust resistance of ‘Rathu Heenati’ at early seedling stages is associated with the effect of *qBPH3.1*. Additionally, a low-virulence BPH population (Hadano-1966) has been advantageous for detecting BPH resistance genes with small effects (Nguyen *et al.* 2021), whereas the use of stronger-virulence BPH populations has been unsuccessful (Jairin *et al.* 2007a). Our study confirmed that it was possible to identify a single resistance gene with less effectiveness against strongly virulent BPH by using Hadano-1966.

In this study, we confirmed the InDel markers co-segregated with *BPH3* and *BPH17* region. The identified co-segregated markers around the gene regions could be efficiently utilized in the MAS for BPH resistance genes in commercial cultivars. Moreover, the significantly improved resistance level of ‘Sagabiyori’ (Saga-*BPH3+17*) demonstrated that the combination of two genes, *BPH3* and *BPH17*, would be effective in enhancing the resistance of commercial Japanese cultivars with heat tolerance. Additionally, the identified *qBPH3.1* with feeding inhibition at early seedling stage might be a comparable factor for the durable resistant of ‘Rathu Heenati’. Therefore, the investigations in the current study would be valuable for the improvement of BPH resistance in commercial Japanese rice cultivars.

## Author Contribution Statement

SBDS, SZ and DF designed the study. SBDS, CDN and DF developed the plant materials. SSM provided BPH populations. SBDS and DF performed the experiments and wrote the paper.

## Supplementary Material

Supplemental Figures

Supplemental Table

## Figures and Tables

**Table 1.** QTL for BPH resistance detected in the BC_1_F_2:3_ population derived from a cross between ‘Taichung 65’ and ‘Rathu Heenati’

QTL	Marker interval	Chr.	LOD score	Phenotypic variance (%)	Additive effect *^a^*	Dominance effect *^a^*
*qBPH3.1*	RM16209–RM16231	3	5.5	26.2	1.01	–0.17

*^a^* The additive and dominance effects indicate the effect of alleles from ‘Taichung 65’.
