# Supplementary material for: Substitution mapping and characterization of brown planthopper resistance genes from traditional rice cultivar ‘Rathu Heenati’ (Oryza sativa L.)
Source: Breed Sci. 2024 Jul 2;74(3):183–92. doi: 10.1270/jsbbs.23066 (PMC11561414; doi:10.1270/jsbbs.23066)
Supplement: Supplementary file 1 — Supplemental Figures [file 74_183-s1.pdf]

Shar et al./Breeding Science
doi: 10.1270/jsbbs.23066

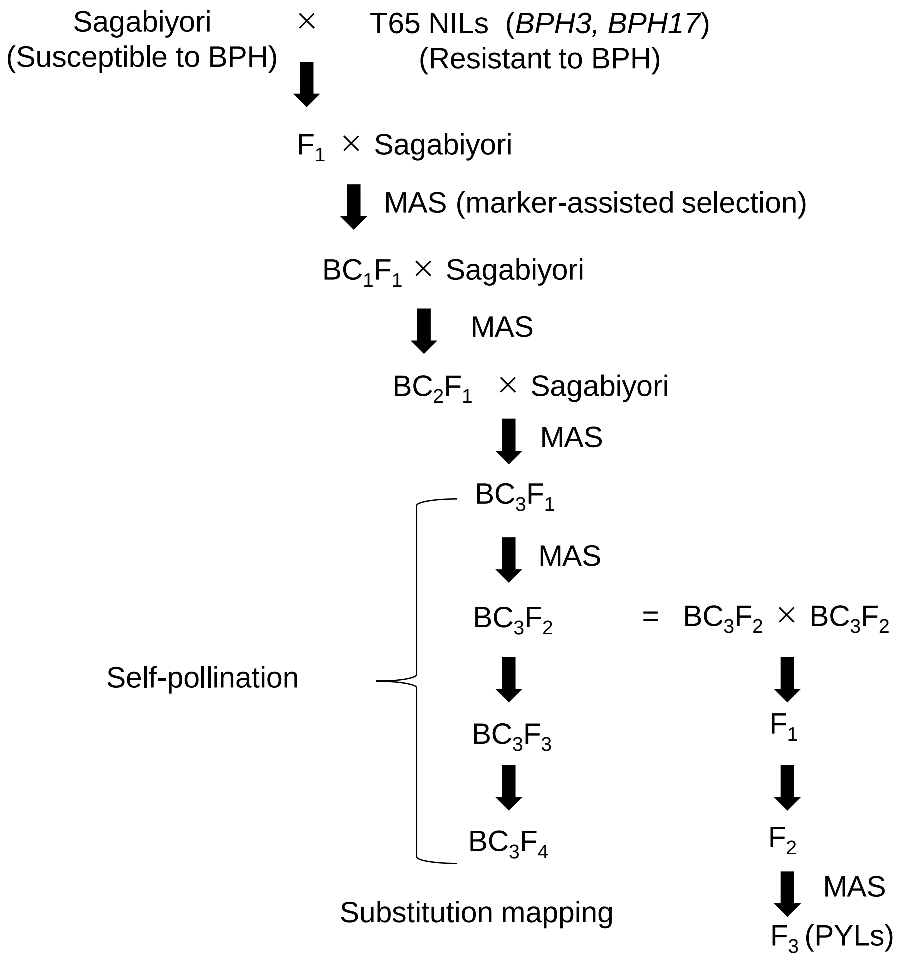

Supplemental Fig. 1. The breeding scheme for the development of substitution mapping populations and pyramided lines for *BPH3* and *BPH17*.

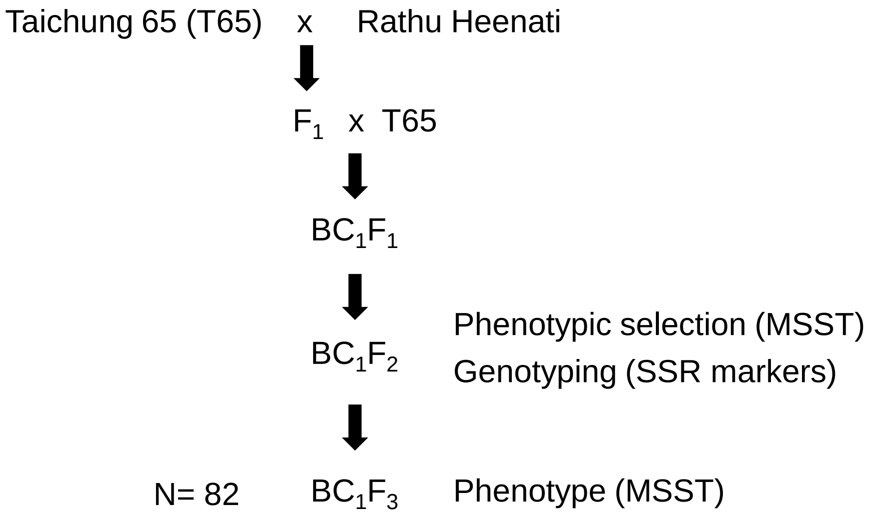

Supplemental Fig. 2. Breeding scheme for development of backcrossed populations for QTL analysis

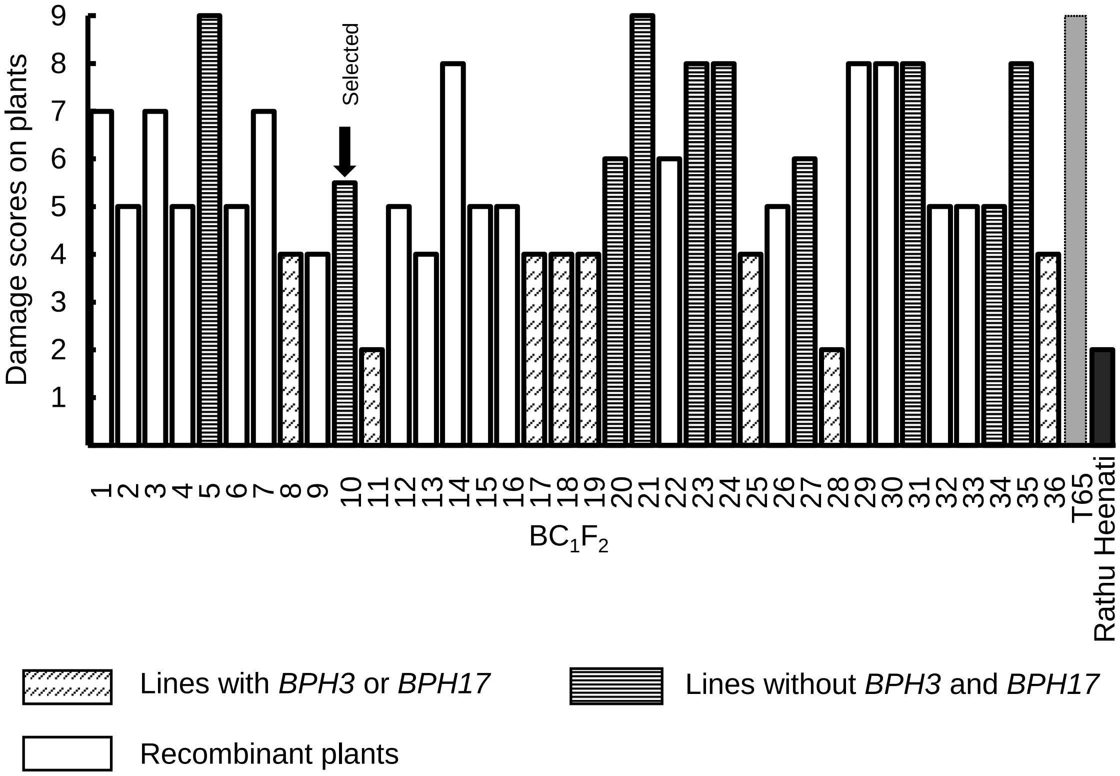

Supplemental Fig. 3. Damage score of 36 BC_1F_2 lines derived from a cross between T65 and 'Rathu Heenati' for population selection.

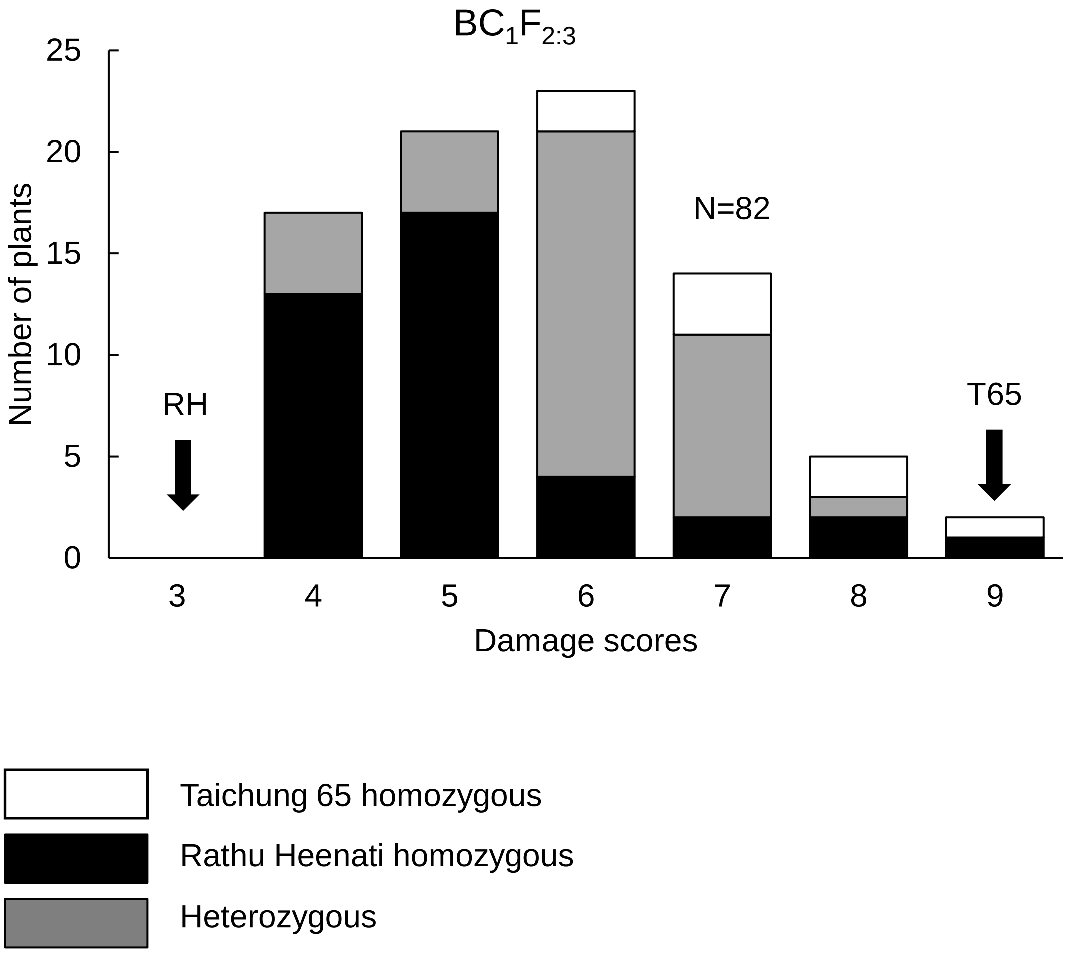

Supplemental Fig. 4. Frequency distribution of damage scores of BPH by MSST and marker genotypes of RM16209 on the $BC_1F_{2:3}$ population derived from a cross between Taichung 65 (T65) and 'Rathu Heenati' (RH).

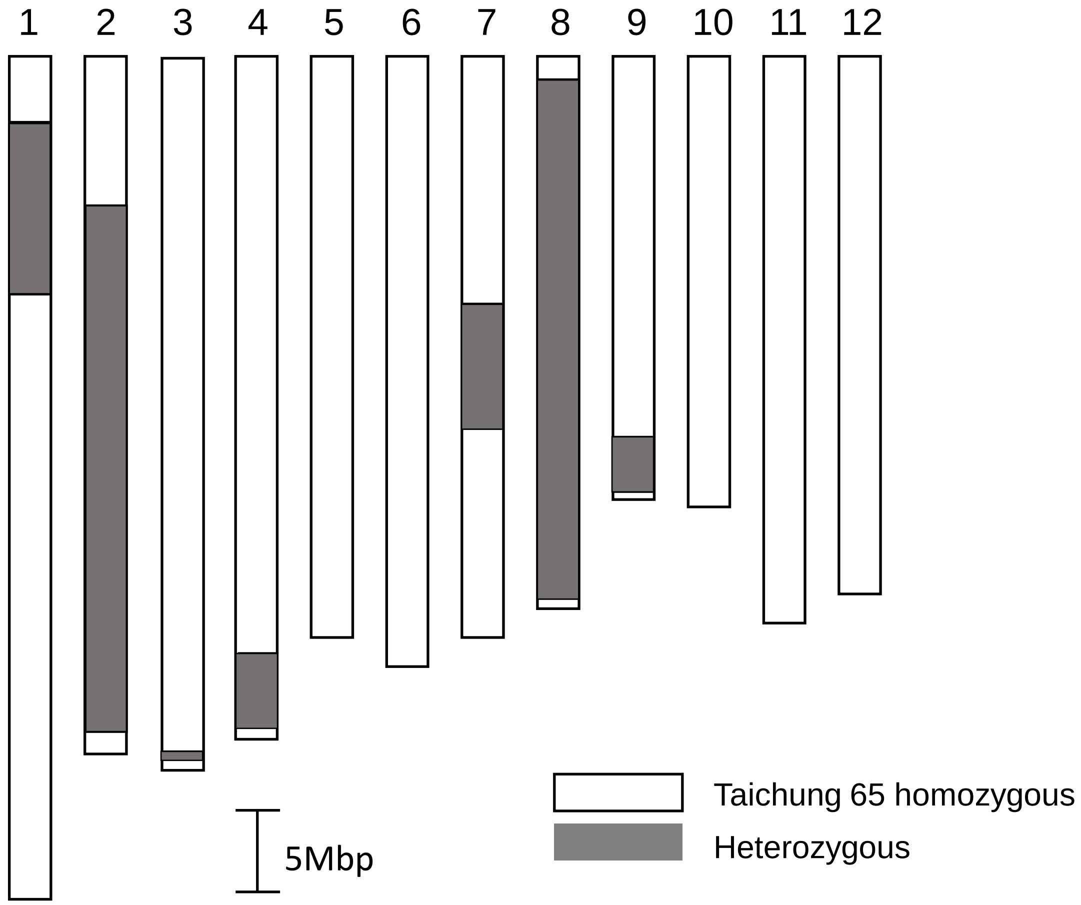

Supplemental Fig. 5. Estimated graphical genotype of BC_1F_1 from the BC_1F_2 bulked plants derived from a cross between 'Taichung 65' and 'Rathu Heenati' using 384 SSR markers. The 12 bars indicate 12 chromosomes of rice.

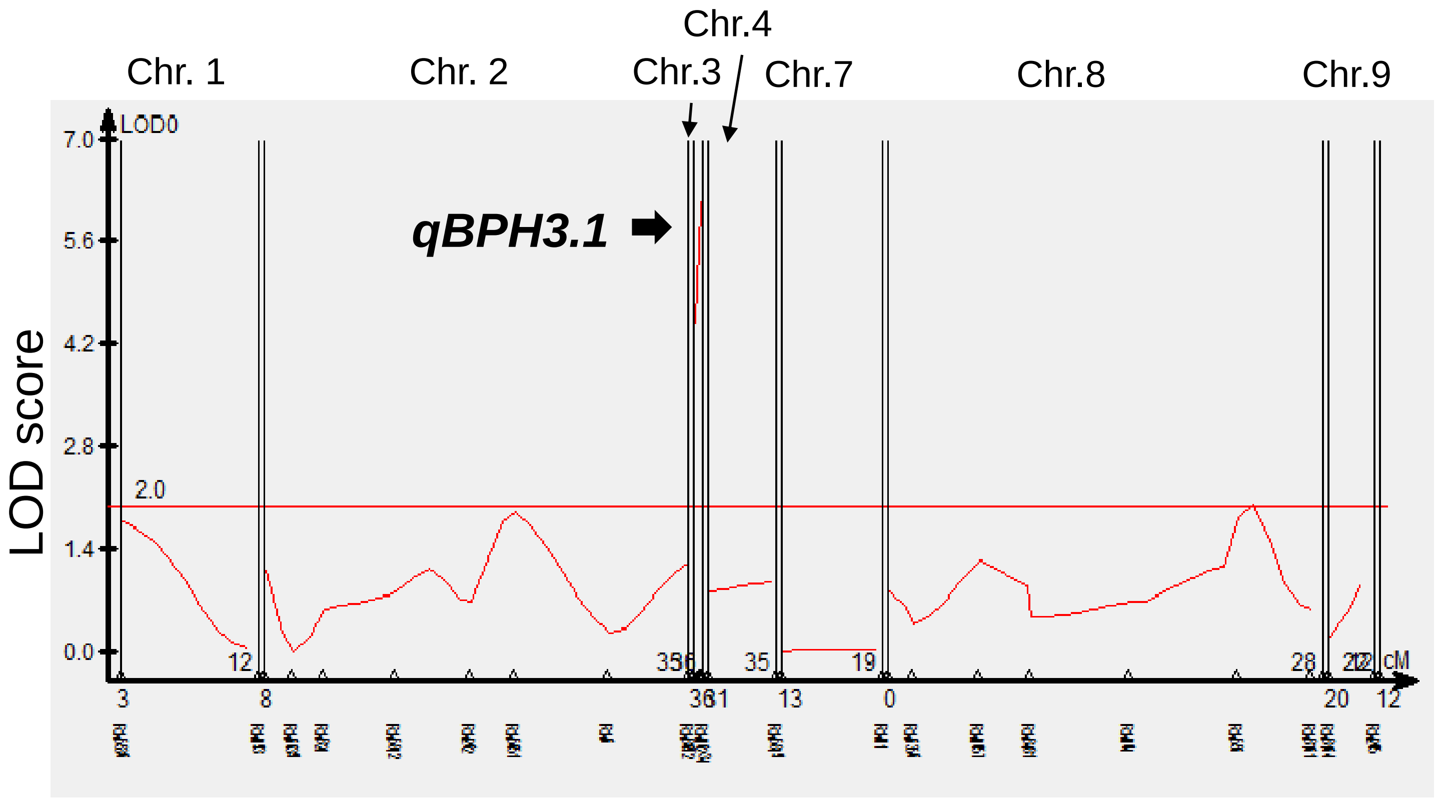

Supplemental Fig. 6. The LOD score of QTL analysis for BPH resistance using $BC_1F_{2:3}$ populations derived from a cross between 'Taichung 65' and 'Rathu Heenati' using Windows QTLs Cartographer v. 2.5.

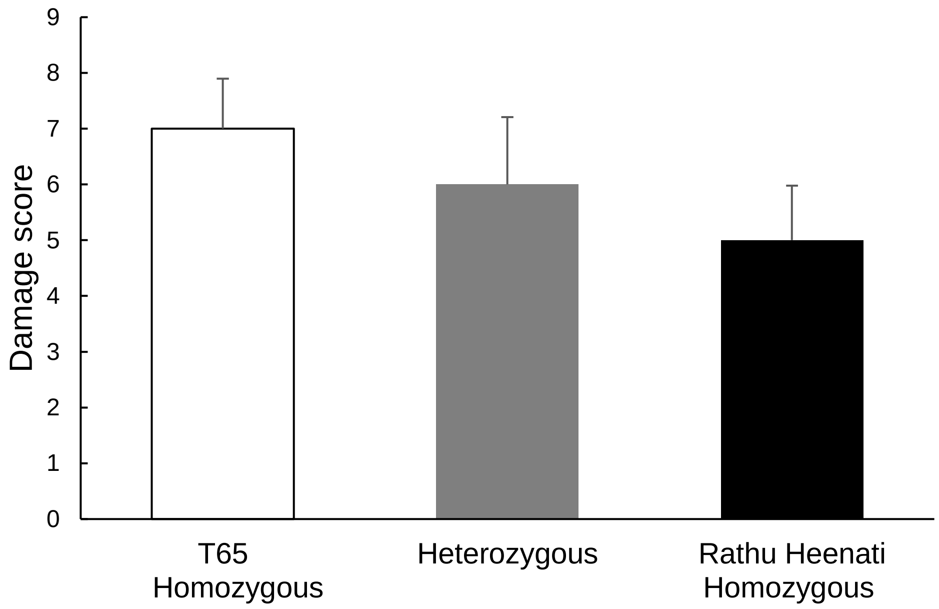

Supplemental Fig. 7. Damage scores of Taichung 65 (T65) homozygous, heterozygous and Rathu Heenati homozygous on the $BC_1F_{2:3}$ population derived from a cross between ‘Taichung 65’ and ‘Rathu Heenati’.

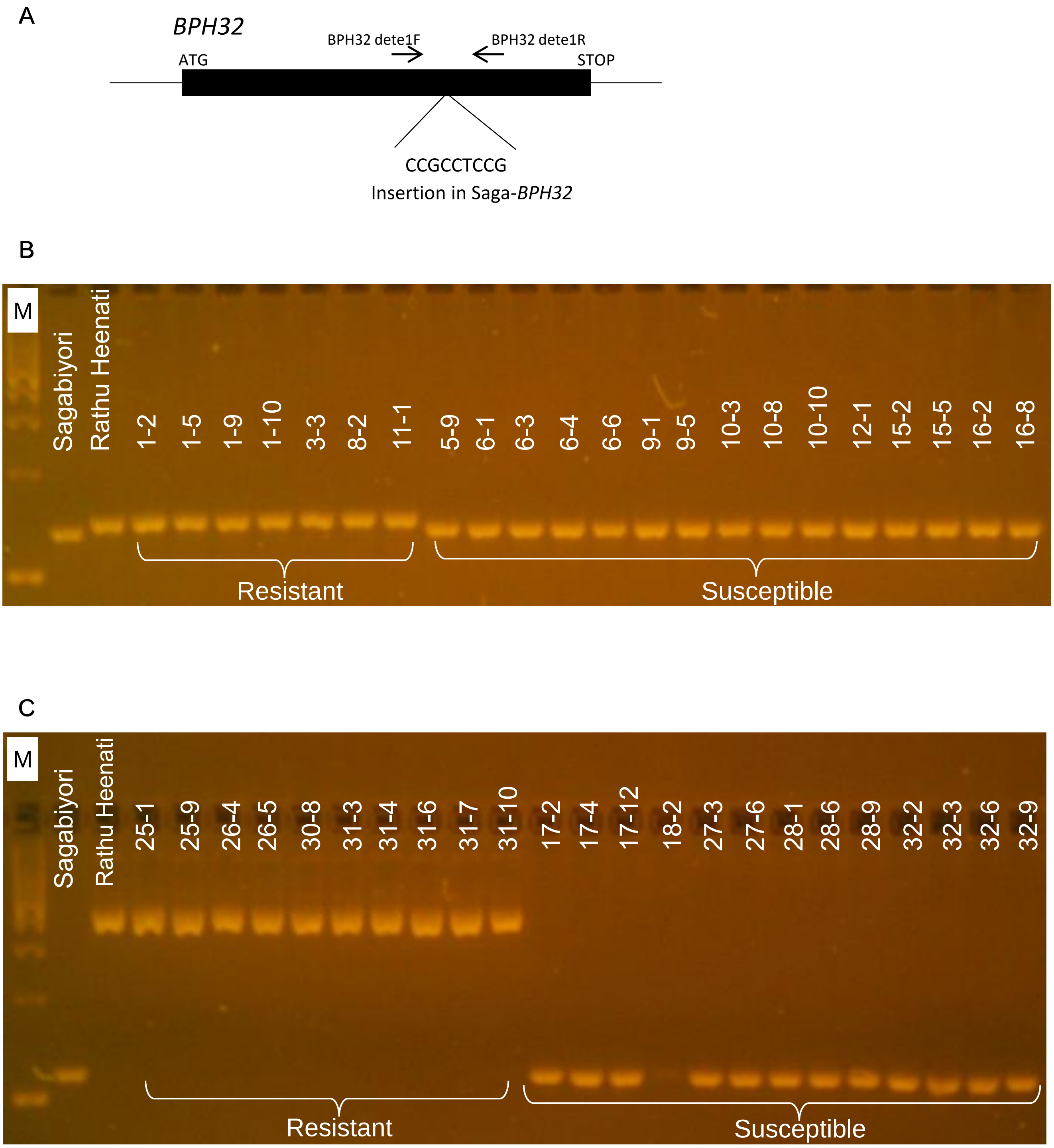

Supplemental Fig. 8. InDel markers (A) structure of BPH32 dete 1, co-segregated (B) BPH32 dete 1 with *BPH3* recombinant lines (BC_3F_4) and (C) I729 with *BPH17* recombinant lines (BC_3F_4) that carried Resistant and Susceptible alleles. M= 100 bp DNA ladder.
