# Supplementary material for: Substitution mapping and characterization of brown planthopper resistance genes from traditional rice cultivar ‘Rathu Heenati’ (Oryza sativa L.)
Source: Breed Sci. 2024 Jul 2;74(3):183–92. doi: 10.1270/jsbbs.23066 (PMC11561414; doi:10.1270/jsbbs.23066)
Supplement: Supplementary file 2 — Supplemental Table [file 74_183-s2.pdf]

doi: 10.1270/jsbbs.23066

Supplemental Table 1. Simple sequence repeat markers used for substitution mapping of *BPH3* and *BPH17*

Marker	Resistance gene tagged	Chr.	Forward Primer sequence (5'-3')	Reverse Primer sequence (5'-3')	Physical location (bp)
RM8213[b]	*BPH17*	4	TGTTGGGTGGGTAAAGTAGATGC	CCCAGTGATACAAAGATGAGTTGG	4,418,222
RM16460[c]	*BPH17*	4	ATTGCACCATTCAAACGGAACC	TTCCAAGCTGTCTTTCTGACATGACC	5,318,612
RM3658[b]	*BPH17*	4	GTAGCACTCCGCTGCTTCGTCTCC	AATCCCACCCGCCTCATCTCC	5,573,675
RM1305[b]	*BPH17*	4	GGTACTACAAAGAAACCTGCATCG	TCCTAGCTCAAATGTGCTATCTGG	5,624,467
RM3471[b]	*BPH17*	4	AGAAACAGAGGGAGGGAGCAGAGG	GATCCCGACAGATGGTGACTTGC	6,279,483
RM16493[b]	*BPH17*	4	TCGGCAGCAACAACTCTTAAACC	TCAACAAGATGACTCCCTGTAGCC	6,375,991
RM16506[c]	*BPH17*	4	GCAGTAGACCTCGTGCTGAATGC	CCACACCGCCGCAATATAAACC	6,926,963
**I531**[e]	*BPH17*	4	ATTTAGGCTCGTCACGGATG	GGGTTGTTGGGAAAAGGAAT	6,936,531
**I729**[e]	*BPH17*	4	CATCGACATTCACACACCCA	TGCATGTCGCTGAATGGATG	6,936,729
RM16508[c]	*BPH17*	4	TTCATTGTCATCGCCTCATTGG	ACAGGTACAGCTGGGTAGAGAGAAGC	6,954,478
RM16531[c]	*BPH17*	4	CAGTGCAGGAACAAGATTCAGG	CATTGCAGTTGGGTTCTATTGG	7,935,067
B40[d]	*BPH17*	4	CAATACCGGATATCTTGACTCC	CGACCACGCTGCCTATATTC	8,214,283
RM6314[b]	*BPH17*	4	CATGTCTGATATTGCGGTTCAGG	TCAAGCCCTGCCCAACTACG	18,627,879
RM471[b]	*BPH17*	4	AGAAATGGATCGGACTGAACATGC	AGACACTCGGACGCACAAGC	19,007,714
RM1205[b]	*BPH17*	4	CAATCACAGAGCAACACGTACCC	GCAGAGGCAGCTGAGAAGTATAGC	19,643,755
RM5586[b]	*BPH17*	4	AGATGGCTGGCCAACAGACTGG	ACAATGCCCATCCACTGCTTCC	19,908,685
RM1359[b]	*BPH17*	4	CGACTTGCCAAAGGTCAACG	GATTCTACGGGCCACAAGTCC	20,041,155
RM6775[b]	*BPH3*	6	AATTGATGCAGGTTCAGCAAGC	GGAAATGTGGTTGAGAGTTGAGAGC	209,054
S00310[d]	*BPH3*	6	CAACAAGATGGACGGCAAGG	TTGGAAGAAAAGGCAGGCAC	214,278
RM508[a]	*BPH3*	6	AGAAGCCGGTTCATAGTTCATGC	ACCCGTGAACCACAAAGAACG	441,752
RM19248[b]	*BPH3*	6	GGAACAACCAGGACTGAGAAGG	CTCGTCACTTGTCAGATCCAACG	467,380
RM19255[b]	*BPH3*	6	TTAAGCTAGGGAATCAGCGGTTAGC	GGAGTTGCAGTGTGGTGTGTGG	534,728
RM469[b]	*BPH3*	6	TTACGTGATCACACAGGCTCTCC	AAGCTGAACAAGCCCTGAAAGG	564,234
RM19262[b]	*BPH3*	6	GGAAAGACCAGTTTAGAGCAATGG	AGCTAGATCCCTTGTTTCACACG	713,147
RM8060[b]	*BPH3*	6	GGTTGTGCTGAATACTGTCCATAAGC	CAGGTAACCGGTGAAGATGTCG	720,892
RM3132[b]	*BPH3*	6	TTTGAGGAGAGTTCTTGGGTTTGG	CTCCACTTCTCTCTTCACTCCCTTCC	799,679
RM19274[b]	*BPH3*	6	CCTGTGAATGACAACCCATGC	GTATGAGCCAGATTAGCGGTTGC	1,010,622
RM19288[c]	*BPH3*	6	CGGAGCTGTTGCCGTTCTGC	CGATGTGCCATGTCAGGATGACC	1,173,479
RM19291[c]	*BPH3*	6	CACTTGCACGTGTCCTCTGTACG	GTGTTTCAGTTCACCTTGCATCG	1,215,950
**BPH32 dete 1**	*BPH3*	6	ACGTATGGGCTGCCTCTATC	TGTAGAGCCTTGTGTGAGCA	1,223,405
RM19296[c]	*BPH3*	6	CTAGCTTGACGCCAAGGACACC	GCACAGACGCACACTGATCTCC	1,290,544
RM589[a]	*BPH3*	6	GTGGCTTAACCACATGAGAAACTACC	TCACATCATTAGGTGGCAATCG	1,380,931
RM19311[c]	*BPH3*	6	TGCGGTGCTGTTCACCTACTATCG	GCACTGAAGCTGGTGCAATCG	1,463,445
RM588[a]	*BPH3*	6	TCTTGCTGTGCTGTTAGTGTACG	GCAGGACATAAATACTAGGCATGG	1,611,442
RM19338[b]	*BPH3*	6	TGGTCTATGGCGTGATTTGTCG	CTAACCACCACGGATCTGAAACC	1,728,980
RM19341[c]	*BPH3*	6	GCTACAAATAGCCACCCACACC	CAACACAAGCAGAGAAGTGAAGC	1,764,661

Primer sequence information was obtained from: [a] Temnykh *et al*. (2001), [b] McCouch *et al*. (2002), [c] International Rice Genome Sequencing Project (IRGSP, 2005), [d] Rahman *et al*. (2009) and [e] He *et al*. (2020). The physical positions of primers for each marker were obtained from The Rice Annotation Project Database (Sakai *et al*. 2013).
